# Exploring bacterial community composition and immune gene expression of European eel larvae (*Anguilla anguilla*) in relation to first-feeding diets

**DOI:** 10.1371/journal.pone.0288734

**Published:** 2023-07-27

**Authors:** Kasun A. Bandara, Elisa Benini, Sebastian N. Politis, Luis E. C. Conceição, André Santos, Sune Riis Sørensen, Jonna Tomkiewicz, Olav Vadstein

**Affiliations:** 1 Technical University of Denmark, National Institute of Aquatic Resources, Lyngby, Denmark; 2 Department of Biotechnology and Food Science, NTNU—Norwegian University of Science and Technology, Trondheim, Norway; 3 SPAROS Lda, Área Empresarial de Marim, Lote C, Olhão, Portugal; Tanta University Faculty of Agriculture, EGYPT

## Abstract

European eel (*Anguilla anguilla*) is a commercially important species for fisheries and aquaculture in Europe and the attempt to close the lifecycle in captivity is still at pioneering stage. The first feeding stage of this species is characterized by a critical period between 20 to 24 days post hatch (dph), which is associated with mortalities, indicating the point of no return. We hypothesized that this critical period might also be associated with larvae-bacterial interactions and the larval immune status. To test this, bacterial community composition and expression of immune and stress-related genes of hatchery-produced larvae were explored from the end of endogenous feeding (9 dph) until 28 dph, in response to three experimental first-feeding diets (Diet 1, Diet 2 and Diet 3). Changes in the water bacterial community composition were also followed. Results revealed that the larval stress/repair mechanism was activated during this critical period, marked by an upregulated expression of the *hsp90* gene, independent of the diet fed. At the same time, a shift towards a potentially detrimental larval bacterial community was observed in all dietary groups. Here, a significant reduction in evenness of the larval bacterial community was observed, and several amplicon sequence variants belonging to potentially harmful bacterial genera were more abundant. This indicates that detrimental larvae-bacteria interactions were likely involved in the mortality observed. Beyond the critical period, the highest survival was registered for larvae fed Diet 3. Interestingly, genes encoding for pathogen recognition receptor TLR18 and complement component C1QC were upregulated in this group, potentially indicating a higher immunocompetency that facilitated a more successful handling of the harmful bacteria that dominated the bacterial community of larvae on 22 dph, ultimately leading to better survival, compared to the other two groups.

## 1. Introduction

Global consumption of aquatic food has increased and is expected to rise continuously in coming years [1]. The sum of fisheries and aquaculture production was 178 million tonnes in 2020, and aquaculture production alone was 87.5 million tonnes. As one of the fastest growing animal production sectors in the world, aquaculture plays an important role in fulfilling the nutritional requirements of the growing human population globally [1]. Marine fishes in particular are a rich source of omega-3 fatty acids, besides protein and minerals [2, 3]. Aquaculture also helps to release the pressure on the natural marine fishery resources [4]. In this regard, it is the closing of the life cycle of fish in captivity that is the basis for development of successful aquaculture, by ensuring reliable, high-quality, and year-round hatchery production of offspring to sustain the commercial farming [5]. Still, unreliable availability of hatchery produced offspring is a constraint for successful marine fish culture, among other, as a sustainable alternative to wild-caught juveniles [6].

European eel (*Anguilla anguilla*) is a marine finfish species of high market value with substantial fisheries and aquaculture potential [7]. However, aquaculture of eels for human consumption and stock enhancement relies entirely on wild juveniles called “glass eels”, which are caught in targeted fisheries [8]. Moreover, the stock has declined sharply, and the European eel is ranked as critically endangered on the IUCN red list [9]. It is therefore imperative to develop hatchery technology for sustainable aquaculture, stock management and conservation plans. Currently, efforts in applied research to establish hatchery techniques and technology have led to a stable production of European eel larvae entering the feeding stage (around 9 dph) [10]. This progress involves assisted reproduction technology and methodology resulting in viable fertilised eggs, their incubation until hatch (~2 days post fertilisation) and development into the feeding stage (10–12 days post hatch (dph)). Present challenges, aiming at establishing protocols for rearing of feeding larvae, include identification of specific nutritional requirements, optimisation of rearing techniques and microbial management in recirculating aquaculture systems (RAS).

For Anguillid eels in general, a major constraint during first feeding of larvae is the establishment of effective diets and adequate feeding regimes. This is mainly due to the limited knowledge about the natural diet of eel larvae during the first-feeding phase and long-lasting migratory phase, from oceanic spawning areas (i.e., the leptocephalus stage) to continental waters, where they metamorphose into glass eels. One enigma of the larval feeding ecology has been that they, in contrast to most other fish larvae, do not appear to feed on zooplankton [11]. Recent studies of leptocephali gut contents suggest that their natural diet consists of amorphous material of different origin, such as larvacean houses, faecal pellets, gelatinous zooplankton, and materials associated with marine snow (bacteria, protists, fungi, and other microorganisms) [11, 12]. This is in sharp contrast to diets that have proven successful for hatchery-reared eel larvae. For first feeding of larval of Japanese eel (*Anguilla japonica*), a species closely related to European eel, a diet based on egg yolk of spiny dogfish (*Squalus acanthias*) was successful [13]. This slurry-type diet sustained larval survival until 26 dph. A gradual modifications and improvements of the diet, together with improved rearing techniques, has enabled closure of the life cycle of Japanese eel and thus production of further generations of captive propagated offspring [14].

For European eel, establishing first feeding larval culture protocol is at a pioneering stage, where successful production of viable larvae and enhanced larval culture technology has increased survival of larvae entering to feeding stage and recently enabled feeding experiments [15, 16]. A recent study showed upregulated expression of genes involved in growth and digestion when pre-fed a slurry type diet based on pasteurized egg yolk from thornback ray (*Raja clavat*a), suggesting a potential benefit of early feeding for improved transition from endogenous to exogenous feeding [17]. Moreover, a further study [18] testing three different slurry-type diets based on spiny dog fish eggs throughout the first feeding window, and documented improved larval growth, survival and digestion, and growth-related gene expression at the end of the experiment (28 dph). Notably, this study represents a landmark as the first documentation of European eel larvae overcoming the bottleneck during the first feeding window and surviving beyond the point of no return. Interestingly, the study revealed two periods of high mortalities: shortly after introduction of feed (10–12 dph) and during the period 20–24 dph. This indicates that besides nutritional (in)appropriateness, other factors such as detrimental larvae-bacteria interactions might be in play.

In marine fish larval culture, negative larvae-bacteria interactions are known to cause low and unpredictable growth and survival [19, 20]. The importance of microbial management during egg incubation and larval culture of European eel has previously been demonstrated, where hatching success and larval longevity were negatively impacted by microbial activity [21]. During feeding with a slurry-type diet, a rapid deterioration of water quality in the rearing tanks is observe [17, 22]. This is likely due to leakage of nutrients to the water and stimulation of microbial growth. In this regard, eel larvae, which in nature thrive in an oligotrophic environment with low bacterial densities [23], might in culture be challenged by detrimental larvae-bacteria interactions caused by the introduction of these easily degradable feeds into the rearing tanks. This challenge may be amplified by a not fully developed larval immune system [24, 25].

In general, newly hatched fish larvae are highly sensitive to detrimental bacteria as their immune system is not fully developed and the intensive rearing causes stress [26]. In marine fish larvae, it can take up to three months until their immune response is fully functional, a long period during which larvae depend largely on the innate part of the immune system and are vulnerable to detrimental interactions and dysbiosis in the microbiota [27, 28]. Since innate immunity plays an important role in protecting fish larvae against microbial interference, larval survival might closely be linked to their immune ontogeny [26]. In fact, a previous study investigating immune gene expression during early life history of European eel, indicated a sensitive phase during which larvae are potentially immuno-compromised [25].

We hypothesized that the 2^nd^ period with increased mortality, described during the first feeding stage of European eels, might be linked to changes in microbial communities and the molecular ontogeny of the immune system. The present study complements the study of [18], by elucidating the impact of the three slurry type diets on the succession of bacterial communities throughout and beyond the first feeding period. Furthermore, the study attempts to disclose the interplay between larvae-bacteria interactions, immune and stress/repair related gene expression and larval performance (survival).

## 2. Materials and methods

### 2.1 Ethics statement

All fish were handled according to the European Union regulations concerning the protection of experimental animals (Dir 2010/63/EU). Experimental protocols were approved by the Animal Experiments Inspectorate (AEI), Danish Ministry of Food, Agriculture and Fisheries (permit number: 2020-15-0201-00768). Broodstock was anaesthetised individually before tagging, biopsy, and stripping of gametes, and euthanised after stripping (females) or at the end of the experiment (males) by submergence in an aqueous solution of ethyl p-aminobenzoate (benzocaine, 20 mg/L, Sigma Aldrich, Germany) [29]. Larvae were anaesthetised and euthanised using tricaine methanesulfonate (MS-222, Sigma Aldrich, Germany) at a concentration of 7.5 and 15 mg/L, respectively [29].

### 2.2 Broodstock husbandry, offspring production and rearing

**Fig 1:**
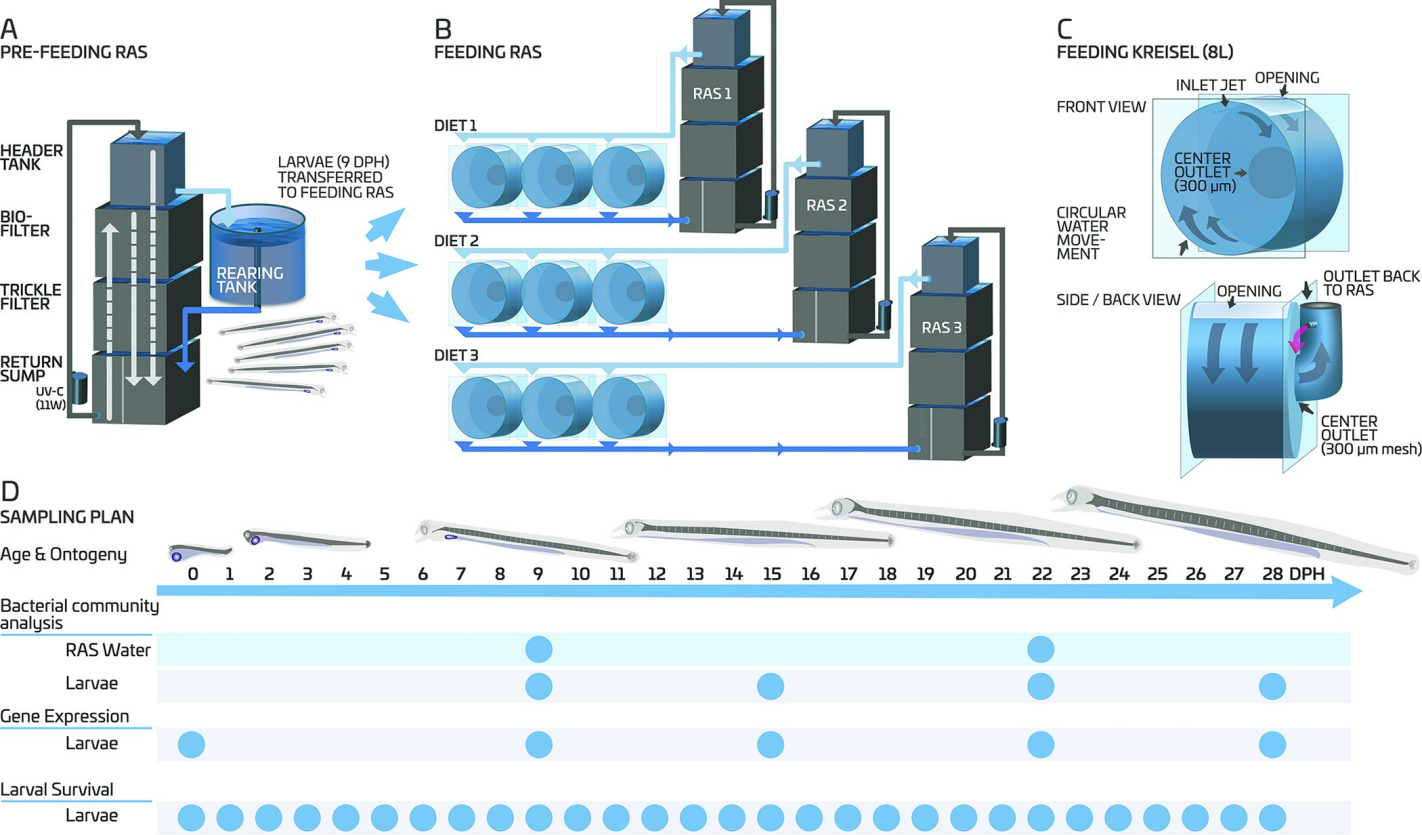
European eel (*A*. *anguilla*) larvae were reared in a common larval rearing tank during the pre-feeding (endogenous feeding) period (from 0 to 9 days post hatch (DPH)) (A) and in 8L acrylic Kreisel tanks (n = 3 for each diet) throughout and beyond the first-feeding window (B and C). Water from the RAS was sampled before (on 9 DPH) after (on 15 DPH) the initiation of the feeding for bacterial community composition analysis and larvae were sampled at different ages for bacterial community composition analysis and molecular analysis of immune and stress related genes, while larval survival was calculated through enumeration of dead larvae daily (D).

The experiment was conducted at EEL-HATCH, Hirtshals, Denmark (57.5858410°N, 9.9853423°E), an experimental hatchery of DTU Aqua. Female European eel broodstock originated from Saltbæk Vig, Zealand, Denmark, while farm-raised males were obtained from a Danish commercial fish farm (Royal Danish Fish A/S, 57.1226075°N, 8.6243243°E). The broodstock was acclimatised to a salinity of 36 PSU and a temperature of 18–20°C. Gametes were obtained through assisted reproduction as described earlier [30]. After fertilisation of eggs using standardised procedures [31], embryos were incubated in 60 L conical bottom incubators supplied with filtered and UV-treated North Sea water adjusted to a salinity of ~36 PSU with Sea Salt (Aquaforest, Brzesko, Poland) [32] and a temperature of ~18°C [33]. At ∼52 hours post-fertilisation (hpf), aeration was stopped, and embryos hatched at ∼56 hpf. Within 6 h after hatching, larvae were transferred to a 77 L tank connected to a 1.7 m^3^ RAS and reared until 9 dph (Fig 1A). During this pre-feeding period, the temperature was maintained at 18–20°C and salinity at ~36 PSU [17]. Water flow was set to 600 mL/min, and rearing was in constant darkness [34].

### 2.3 Experimental design and implementation

The present study is part of a feeding experiment in which various aspects of larval development and culture conditions were investigated and related to larval survival. The present study focuses on the succession of bacterial communities and larval immune gene expression, whereas another study targeted the effect of diets on larval appetite, feeding success and growth as well as related gene [18].

For the experiment, a batch of larvae was selected on 9 dph. Larvae were stocked in replicated (n = 9) 8 L Kreisel tanks at a density of ~60 larvae/L. These nine Kreisel tanks were randomly assigned in triplicates to three different experimental groups based on the type of diet (i.e., Diet 1, Diet 2, and Diet 3; three tanks per diet) and connected to three separate RAS units (one RAS per diet) (Fig 1B and 1C). In total we used: 3 diets × 3 reps × ∼60 larvae/L × 8 L = ∼4320 larvae. The RAS units, maintained at a salinity of 18 PSU [35] and temperature of 20°C [33], were identical in terms of design. Each was composed of an upper sump reservoir of 370 L, which housed an 80 L wet/dry trickling filter, filled with RK bio-elements (240 m^2^ surface area or 0.12 m^2^ per L), a lower sump reservoir (260 L), a protein skimmer (Aquamedic 5000 single 6.0, Bissendorf, Germany) and UV treatment (ProCristal UV-C 11W, JBL GmbH & Co. Neuhofen, Germany). Each system was connected to an extra reservoir of 160 L to create head pressure before reaching the rearing tanks. Flow rates into the tanks were kept at ~ 420 mL/min, i.e., a hydraulic retention time of 0.32 h, except during feeding. Light (~500 lux) was turned on only during feeding [15].

Throughout the feeding period, water quality parameters were measured regularly and maintained within the optimum range. Temperature and salinity were 20 ± 0.5°C and 18 ± 0.4 PSU, respectively. The range of dissolved oxygen (DO) during the experiment was within acceptable levels. DO levels at the end of feeding (after flow has been stopped for 30 mins to allow larvae to settle to bottom and eat) was 6.5 ± 0.2 mg O_2_/L. When the water flow was on, DO level was 7.8 ± 0.1 mg O_2_ /L. Water pH was 8 ± 0.2. The toxic nitrogen components in water (NH_4_^+^/NH3 and NO_2_^-^), which were measured with standard test kits were below the detection limits.

The proximal compositions of the three diets applied during the experiment are given in Table 1 and explained in detail previously [18]. Diet 1, which consisted of pasteurised spiny dogfish (*S*. *acanthias*) egg yolk (Sterna Seafood AS, Snarøya, Norway), krill extract, soybean peptides and water, contained less protein and more lipids compared to the other diets. Diets 2 and 3 represented modifications of Diet 1, where spiny dogfish (*S*. *acanthias*) egg yolk was partially substituted with hydrolysed proteins. Diet 2 contained fish hydrolysate (CPSP90, Sopropeche, France) encapsulated in whey (Volacactive UltraWhey 80 Instant, Volac International Ltd, Hertfordshire, UK), whereas Diet 3 contained only whey. The diets were comparable in energy content.

**Table 1:** Composition of the three diets used during the experiment (see also [18]).

	Diet 1	Diet 2	Diet 3
Dry matter (%)	27.1 ± 0.12	29.8 ± 0.09	36.01 ± 0.10
Protein (%)	50.89 ± 0.35	61.12 ± 0.17	59.09 ± 0.81
Lipid (%)	37.52 ± 0.25	27.49 ± 0.10	27.51 ± 0.28
Ash (%)	3.33 ± 0.04	3.21 ± 0.12	2.91 ± 0.03
Energy (kJ/g)	29.30 ± 0.18	27.93 ± 0.17	28.46 ± 0.18

The different experimental groups were fed with their respective diet five times per day at 2 h intervals. Before feeding, lights in the larval rearing room were turned on and water flow to the tanks was stopped. The lights were programmed to start with a low intensity and gradually increase the intensity to minimise the stress caused by exposure to light. Then, diets were pipetted on the bottom of the tank at a concentration of 0.5 mL/L of water. After allowing the larvae to feed for 30 min, lights were turned off and the water flow was started. The remaining food on the tank bottom was flushed with a gentle jet of water. Water in the rearing tanks flowed through for 30 min (by disconnecting the tanks from the rest of the recirculating unit) before the tanks were reconnected to each corresponding RAS. To compensate for the loss of water, each RAS unit was refilled by adding water pre-adjusted to 20°C and 18 PSU. The larvae were moved into clean tanks each day [17].

### 2.4 Sampling and data collection

#### 2.4.1 Larval survival

Larval survival was monitored daily during the exogenous feeding period through counting and removing dead larvae. Additionally, all larvae at the end of the experiment (28 dph) as well as those sampled from each experimental unit were enumerated. Larval survival was then calculated as a percentage from 9 until 28 dph (Fig 1D).

#### 2.4.2 Characterisation of bacterial community composition by amplicon sequencing

For characterisation of the bacterial community associated with larvae, pools (n = 4) of ~10 larvae were sampled from the common larval rearing tank before the onset of exogenous feeding (9 dph) (Fig 1D). Also, at two intermediate time points during the first feeding period (on 15 and 22 dph), pools (n = 2) of ~10 larvae from each replicate (n = 3) of the experimental groups (n = 3) were collected for microbiome analysis. At the end of the experiment (28 dph), larval samples were collected for microbiome analysis only from the groups fed with Diets 2 and 3. Sampled larvae were immediately euthanised, rinsed and stored at -20°C for later analysis. To investigate whether the three different experimental groups were exposed to a similar initial water microbiome, water samples (n = 4) from each RAS (n = 3) were collected (from the inlet tubes that supplied water to the rearing tanks). To check whether the feeding caused a shift in the water microbiome, water samples (n = 4) from each RAS (n = 3) were collected on day 22 post larval hatch. Here, 250 mL of water from each sample was vacuum filtered through 0.22 μm white gridded filters (diameter = 47 mm; Merck KGaA, Darmstadt, Germany) using a Büchner funnel and the filters were stored in sterile cryotubes stored at −20°C until processing [36].

DNA from larvae (pools of 10 whole larvae) and water were isolated using the MagAttract PowerSoil Pro DNA Kit (Qiagen, Germany) following the protocol developed by the supplier for automated high-throughput isolation of DNA with the Thermo Scientific® KingFisher® Flex platform. Briefly, samples (pools of ~10 larvae, or filter papers) were homogenised in bead-beating tubes containing ~0.55 g of 0.1 mm glass beads (Bertin Technologies, France) and 800 μL of lysis buffer, using a Precellys 24 tissue homogeniser (Bertin Technologies, France) at 5500 rpm for two times 30 s with a 15 s break in between. The tubes containing the lysates were centrifuged at 15000 × g for 1 min and the supernatants transferred into 1.5 mL Eppendorf tubes. Then, 300 μL of CD2 solution was added to each Eppendorf tube, vortexed to mix and centrifuged at 15000 × g for 1 min. Prepared lysates i.e., supernatants from the previous step, were transferred to the KingFisher Flex platform (Thermo Fisher Scientific), where total genomic DNA was captured on specialised magnetic beads in the presence of buffers, washed on the beads and then eluted.

The V3 and V4 regions of the bacterial 16S rRNA gene were amplified from the DNA isolates using the forward primer, Ill-341F_K1 (5′- NNNNCCTAC GGGNGGCWGCAG -3′) and the reverse primer, Ill805R (5′- NNNNGACTACNVGGGTATCTAAKCC-3′) [37]. Each PCR reaction contained 0.02 U/μL Phusion Hot Start II DNA polymerase (Thermo Scientific), 0.2 mM of each dNTP (VWR), 0.3 μM of each primer (SIGMA), 1x Phusion HF buffer (containing 7.5 mM MgCl2) (Thermo Scientific) and PCR grade water (VWR) up to a total reaction volume of 25 μL, and 1 μL of DNA extract as a template. The PCR reactions were run with 35 cycles (T100TM Thermal Cycler, Bio-Rad) [38]. The PCR amplicons were purified and normalized using SequalPrep Normalization Plate (96) kit (Invitrogen, USA), following the protocol provided by the supplier. Using the Nextera®XT DNA Sample Preparation Kit (Illumina), a unique pair of index sequences that represented the PCR amplicons, originating from each sample, was added by an additional PCR step with 10 cycles. The indexed PCR products were purified and normalized using the SequalPrep Normalization Plate (96) kit (Invitrogen, USA). Finally, the samples were pooled and concentrated with AmiconUltra 5.0 Centrifugal Filter (Merck Millipore, Ireland) following the manufacturer’s protocol. The amplicon library was sequenced in a MiSeq run (Illumina, San Diego, CA) with V4 reagents (Illumina) at the Norwegian Sequencing Centre (NSC), University of Oslo.

The Illumina sequencing data were processed using USEARCH (version 11) (https://www.drive5.com/usearch/). Merging the paired reads, trimming off primer sequences and filtering out reads shorter than 380 base pairs were carried out using the command Fastq_mergepairs. The Fastq_filter command (with an expected error threshold of 1) was used for further processing, which included the steps, demultiplexing, removal of singleton reads, and quality trimming. Unoise3 command was used for chimaera removal and generation of amplicon sequence variants (ASVs) (https://drive5.com/usearch/manual/cmd_unoise3.html). Taxonomy was assigned by applying the SINTAX script [39] with a confidence value threshold of 0.8 against the RDP reference data set (version 18). Before analysing the data, ASVs representing eukaryotic amplicons (e.g., algae, fish DNA), Archaea and Cyanobacteria/Chloroplast were removed from the ASV table. Moreover, the ASVs that were highly abundant in the DNA extraction kit blank and reported as common contaminants were removed. ASVs of special interest were further investigated with the SeqMatch tool to find the matches of those DNA sequences at the RDP website (https://academic.oup.com/nar/article/42/D1/D633/1063201).

#### 2.4.3 Analysis of expression of immune and stress-related genes

For analysis of immune- and stress-related gene expression, larvae were collected during the endogenous (at hatch and 9 dph) and exogenous feeding periods (15, 22, and 28 dph) (Fig 1D). During the endogenous feeding period, three samples, each containing ∼10 larvae were collected from the common larval rearing tank, where the larvae were housed before moving into the different experimental groups. On 15 and 22 dph, pooled samples of ∼10 larvae were randomly collected from each replicate (n = 3) of the three experimental groups. On 28 dph, sampling for molecular analysis was possible only for Diet 3, as not enough larvae were available for sampling for the other two diets. Sampled larvae were immediately euthanised, preserved in RNAlater (Sigma-Aldrich St Louis, USA), and stored at -20°C until analysis [33].

Total RNA from samples was extracted using the NucleoSpin (Mini) RNA isolation kit, following the protocol provided by the supplier (Macherey-Nagel GmbH & Co. KG, Düren, Germany). RNA concentration (110 ± 43 ng/mL) and purity (260/280 = 2.09 ± 0.03, 230/260 = 2.02 ± 0.12) were determined through spectrophotometry using Nanodrop ND-1000 (Peqlab, Germany) and normalized to 100 ng/mL with HPLC water. From the resulting total RNA, 450 ng was reverse transcribed using the qScriptTM cDNA Synthesis Kit (Quantabio, Germany) according to the manufacturer’s instructions, including an additional gDNA wipe-out step before transcription [PerfeCtaR DNase I Kit (Quantabio, Germany)].

Expression levels of 4 target and 3 reference genes were determined by quantitative real-time PCR (qRT-PCR). Primers were designed using primer 3 software v 0.4.01 based on sequences available in Genbank databases (Table 2). All primers were designed for an amplification size ranging from 75 to 200 nucleotides. Expression of genes in each larval sample from each tank (n = 3), diet (n = 3), and larval age (n = 5) were analysed in technical replicates (n = 3) of each gene using the qPCR Biomark^TM^ HD system (Fluidigm) based on 96.96 dynamic arrays (GE chips). In brief, a pre-amplification step was performed with a 500 nM primer pool of all primers in TaqMan-PreAmp Master Mix (Applied Biosystems) and 1.3 mL cDNA per sample for 10 min at 95°C; 14 cycles: 15 s at 95°C and 4 min at 60°C. Obtained PCR products were diluted at 1:10 with low EDTA-TE buffer. The pre-amplified products were loaded onto the chip with SSofast-EvaGreen Supermix low Rox (Bio Rad) and DNA-Binding Dye Sample Loading Reagent (Fluidigm). Primers were loaded onto the chip at a concentration of 50 mM. The chip was run according to the Fluidigm 96.96 PCR protocol with a Tm of 60°C.

**Table 2:** Oligos used for molecular analysis of immune- and stress-related gene expression.

Function	Gene name	Abbreviation	Primer sequence (FW: Forward, RV: Reverse)		Accession no.
Reference	40S ribosomal S18	*rsp18*	FW	AGAGCAGGGGAACTGACTGA	XM_035428800.1
			RV	ACCTGGCTGTATTTGCCATC	
	Tubulin β	*tubb*	FW	TGATGAGCACGGTATTGACC	XM_035419873.1
			RV	TGGCACATACTTTCCACCAG	
	Elongation Factor 1a	*ef1a*	FW	CTGAAGCCTGGTATGGTGGT	XM_035428274.1
			RV	CATGGTGCATTTCCACAGAC	
Complement system	Complement component 1, Q subcomponent, C Chain	*c1qc*	FW	TCTGCTGTCATGTTCACCCA	XM_035433127.1
			RV	CTTCTCGCCATCCCTTCCAT	
Pro-inflammatory Cytokines	Interleukin 1β	*il1b*	FW	ATTGGCTGGACTTGTGTTCC	XM_035380403.1
			RV	CATGTGCATTAAAGCTGACCTG	
Pathogen recognition	Toll like receptor 18	*tlr18*	FW	TGGTTCTGGCTGTAATGGTG	XM_035421803.1
			RV	CGAAATGAAGGCATGGTAGG	
Stress/ repair	Heat shock protein 90	*hsp90*	FW	ACCATTGCCAAGTCAGGAAC	XM_035392491.1
			RV	ACTGCTCATCGTCATTGTGC	

The genes 40S ribosomal S18 (*rps18)*, tubulin β (*tubb*) and elongation factor 1a (*ef1a*) were chosen as reference genes, as they have been suggested to be the most stable in fish larvae and thus, the most reliable reference genes [40]. Their stability was statistically confirmed, as their expression was not significantly different across treatments. The relative quantity of target gene transcripts (ΔCT) was normalized to the geometric mean of the three reference genes chosen above. The coefficient of variation (CV) of technical replicates was calculated and checked. Further analysis of gene expression was carried out according to the 2^-ΔΔCt^ method [41] to calculate the expression of targeted genes relative to the levels at hatch.

### 2.5 Statistical analysis

#### 2.5.1 Measures of microbial diversity

Different packages developed for R statistical software (version 4.2.0) were used to calculate diversity and perform statistical analyses. Alpha-diversity measures including estimated ASV richness (Chao-1) [42], observed ASV richness and evenness, were calculated using the vegan community ecology package (version 2.6.2). Observed ASV richness and evenness were analysed using a series of mixed model ANOVAs. Residuals were evaluated for normality and homoscedasticity (plot of residuals vs. predicted values) to ensure that they met model assumptions. Data were transformed appropriately to meet these assumptions when necessary. Beta-diversity analyses were performed on the ASV table that had been filtered to remove any ASVs that had less than 2 counts in at least two samples and rarefied by sub-sampling ten times at 11503 reads per sample (the threshold was chosen based on the sample with the lowest number of reads). Ordination by principal coordinate analysis (PCoA, 999 permutations) based on Bray–Curtis [43] and Sørensen–Dice dissimilarity was used to visualise differences in microbial community composition between different samples using the function plot_ordination within the phyloseq package (version 1.40.0). Permutational multivariate analysis of variance (PERMANOVA) [44] based on the Bray-Curtis and Sørensen–Dice dissimilarities were used to test for differences in community composition (beta diversity) as a function of diet and age. Pairwise differences were tested using the function pairwise.adonis2 in vegan package (version 2.6.2). The package DESeq2 (version 1.36.0) was used on the unrarefied ASV table to assess the differential abundance of ASVs between the samples that were found to be significantly different by PERMANOVA. DESeq2 includes a model based on the negative binomial distribution and Wald’s post hoc test for significance testing. The P-values adjustment method used was the Benjamin and Hochberg method [45], which accounts for multiple comparisons.

#### 2.5.2 Expression of immune and stress-related genes and larval survival

R studio statistical analysis software (version 4.2.0) was used to perform all the statistical analyses. Residuals were evaluated for normality and homoscedasticity (plot of residuals vs. predicted values) to ensure that they met model assumptions. Data were transformed appropriately to meet these assumptions when necessary. Alpha was set at 0.05 for testing the main effects and interactions. Larval survival and gene expression data were analysed using a series of mixed model ANOVAs, where the main model variables were treatment (Diet 1, Diet 2, and Diet 3) and age, whereas replicated tanks were considered random. The initial model tested included an interaction effect between treatment and age. The model was reduced, when possible, validated through analyses of the residuals, and treatment means were contrasted using Tukey’s honestly significant difference test (Tukey’s HSD).

## 3. Results

### 3.1 Effect of type of diet on larval survival

**Fig 2:**
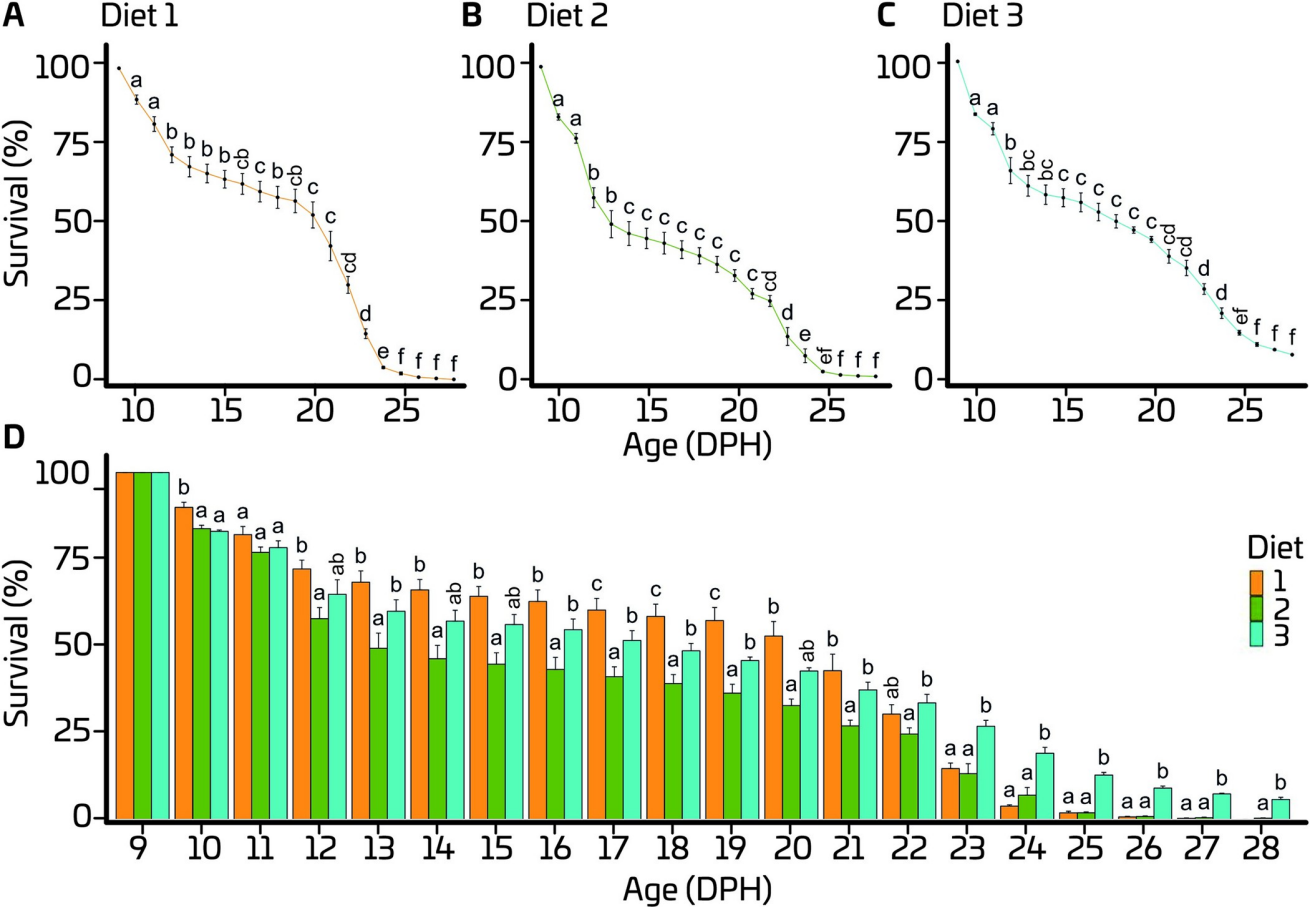
Effect of age for each diet (A-C) and effect of diet at each day-post-hatch on survival of European eel (*A*. *anguilla*) larvae. Values represent means (± SEM), while different lower-case letters represent significant differences (p < 0.05).

Independent of the diet, survival significantly decreased over time. An initial sharp drop was observed during the first 3 days after initiation of feeding, followed by a period with relatively stable survival from 13 to 19 dph (Fig 2A–2C). Thereafter, a second sharp drop in survival occurred between 20 and 24 dph. A significant age × diet interaction was detected for the survival. Thus, the model was decomposed into a series of reduced one-way ANOVAs to analyse the survival as a function of age for each diet and to determine the effect of diet at each day-post-hatch; see also [18]. Diets 1 and 3 equally outperformed Diet 2 until 16 dph, while from 17 to 19 dph, highest survival was observed in the larvae fed Diet 1 (Fig 2D). However, beyond 22 dph, highest survival was observed for larvae fed Diet 3 with a 4% survival at the end of the experiment.

### 3.2 Bacterial community composition analysis

#### 3.2.1 Alpha diversity

**Fig 3:**
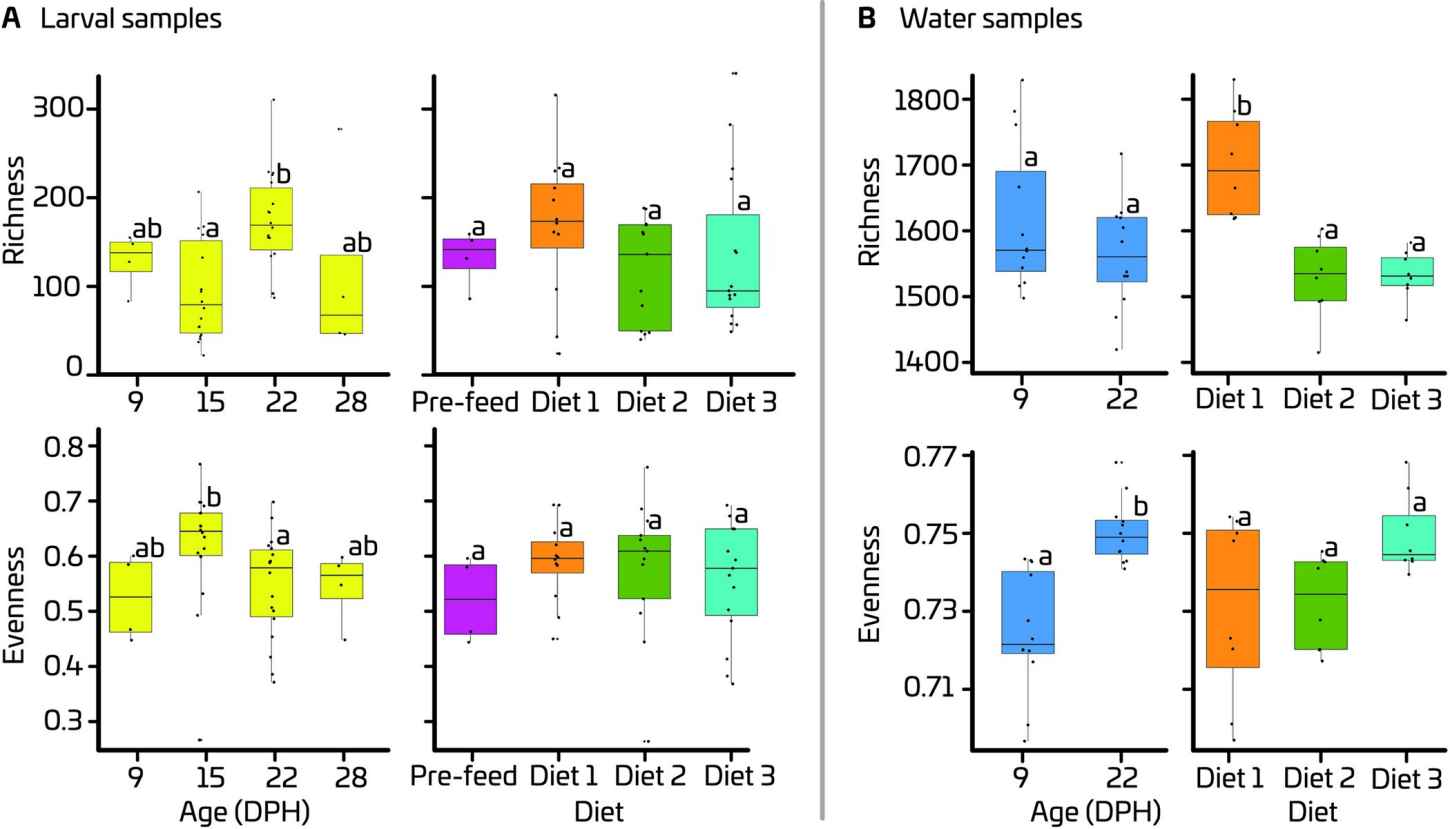
Effects of diets and age (Days Post Hatch (DPH)) on richness and evenness of ASVs in European eel (*A*. *anguilla*) larvae (A) and inflowing water (B). Yellow and blue colours indicate the larval and water samples, respectively. In each box plot, the solid black line indicates mean alpha diversity measure, and the surrounding box indicates the third quantile.

Both ASV richness and evenness in inflowing water were significantly higher than in larvae on both, 9 and 22 dph, regardless of the diet used. There was no significant age × diet interaction for the alpha diversity indices, neither for larval nor for inflowing water samples. For the larval samples, both richness and evenness were affected significantly by age, but not by diet. Richness was 68% higher (p = 0.034) on 22 dph compared to 15 dph (Fig 3A). On the other hand, evenness was 14% lower (p = 0.042) on 22 dph compared to 15 dph. The richness in the inflowing water of the RAS allocated to Diet 1 was 11% higher (p = 0.001) than in the inflowing water of the other two RAS units (Fig 3B). There was no effect of age on the richness of ASVs in inflowing water. Whereas diet had not significantly affected evenness in inflowing water, a significant effect of age was detected, with 3% higher evenness on 22 dph than on 9 dph (Fig 3B).

**Fig 4:**
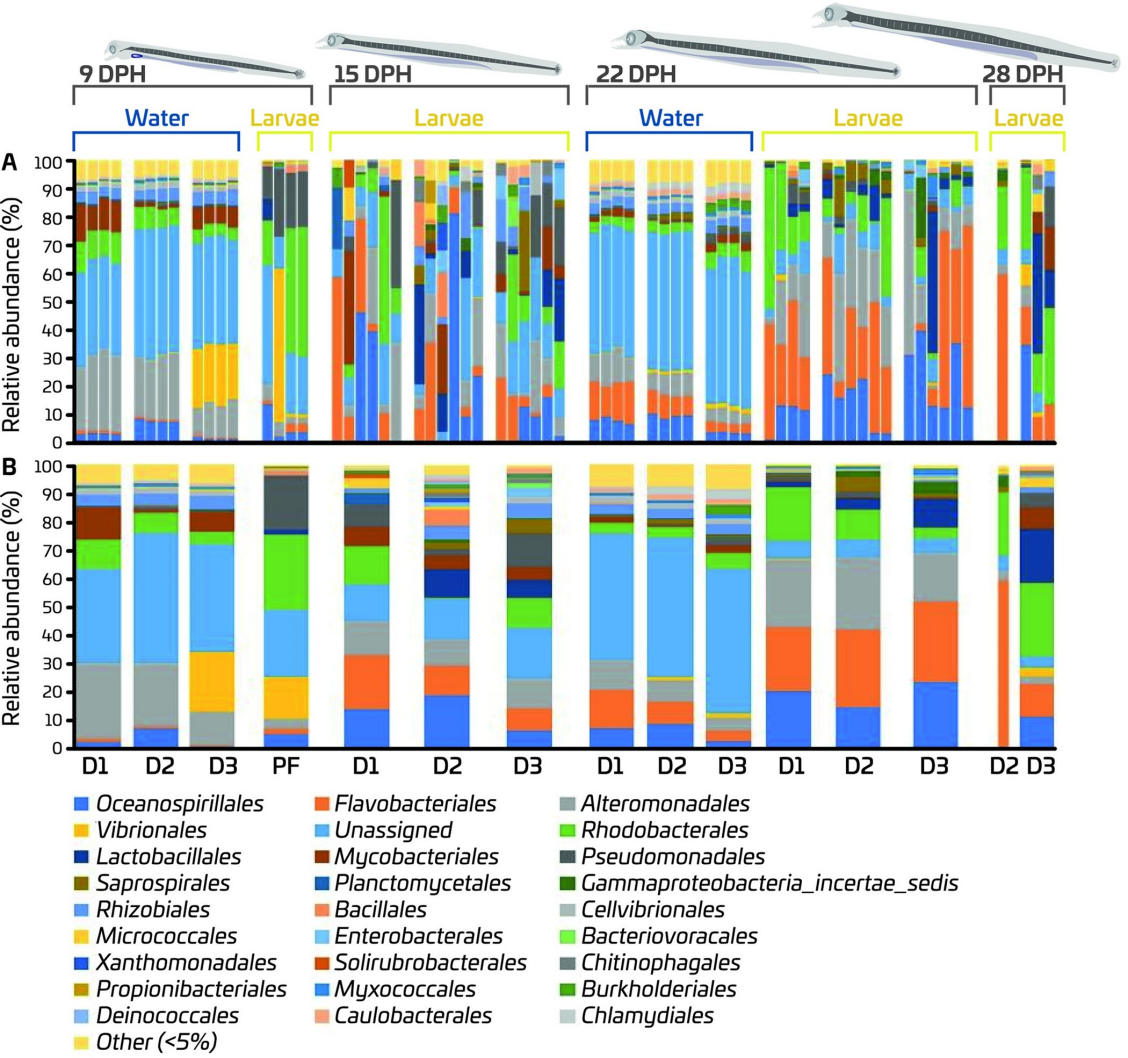
Relative abundances of the bacterial orders detected in European eel (*A*. *anguilla*) larvae as a function of feed and age, and in inflowing water from corresponding RAS units. Each stacked bar represents the relative abundances of bacterial orders detected in each replicate sample (A) and the average of relative abundances in replicate samples (B). Unassigned is ASVs that could not be classified reliably at the order level. (D1 = Diet 1, D2 = Diet 2, D3 = Diet 3, PF = Pre-feeding).

On 9 dph, samples from inflowing water in all three RAS units were dominated by the two orders “unassigned” (>33.4%) and Alteromonadales (>11.9%) (Fig 4). Orders Rhodobacterales, Rhizobiales, Mycobacteriales, Oceanospirillales, Cellvibrionales, and Flavobacteriales were also detected in inflowing water on 9 dph, but at low abundances (<4.6%). Notably, in the inflowing water of the RAS allocated to Diet 3, bacteria belonging to the Vibrionales order, showed a higher relative abundance (20.9%) than in inflowing water of the other two RAS units. On 22 dph, the contribution of “unassigned” (>44.4%), Flavobacteriales (>3.4%) and Oceanospirillales (>3%) orders to the bacterial community in the rearing water was increased, and the share of Alteromonadales (<10.5%) was reduced compared to 9 dph. The abundance of Vibrionales was also decreased in inflowing water of the RAS allocated to Diet 3 on 22 dph compared to 9 dph. On the other hand, the larval bacterial community on 9 dph was dominated mainly by the Rhodobacterales (26.6%), “unassigned” (24%), Pseudomonadales (18.7%) and Vibrionales (14.3%) orders. A shift in the larval bacterial community composition was observed from 15 dph to 22 dph, independent of the diet fed. On 15 dph, a more even larval bacteria community, where “unassigned” (>13.1%), Alteromonadales (>9.6%), Flavobacteriales (>8%), Oceanospirillales (>6.4%), Mycobacteriales (>4.5%) and Rhodobacterales (>0.4%) orders were abundant in all dietary groups. On 22 dph, the abundances of the orders Flavobacteriales (>22.2%), Alteromonadales (17.2%) and Oceanospirillales (14.2%) have increased comprising more than 60% of larval bacterial community composition independent of the diet. On 28 dph, larval samples were not available for Diet 1. For Diet 2, one larval sample was available, and the bacterial community of this sample was dominated by the orders, Flavobacteriales (59.2%) and Rhodobacterales (22.4%). For Diet 3, again a more even bacterial community was detected on 28 dph, where contribution of Rhodobacterales (25.7%), Lactobacillales (19.0%) and Mycobacteriales (7.6%) was increased, while contribution of Flavobacteriales (11.7%), Oceanospirillales (11.4%), and Alteromonadales (2.5%) was decreased compared to 22 dph.

#### 3.2.2 Beta diversity

**Fig 5:**
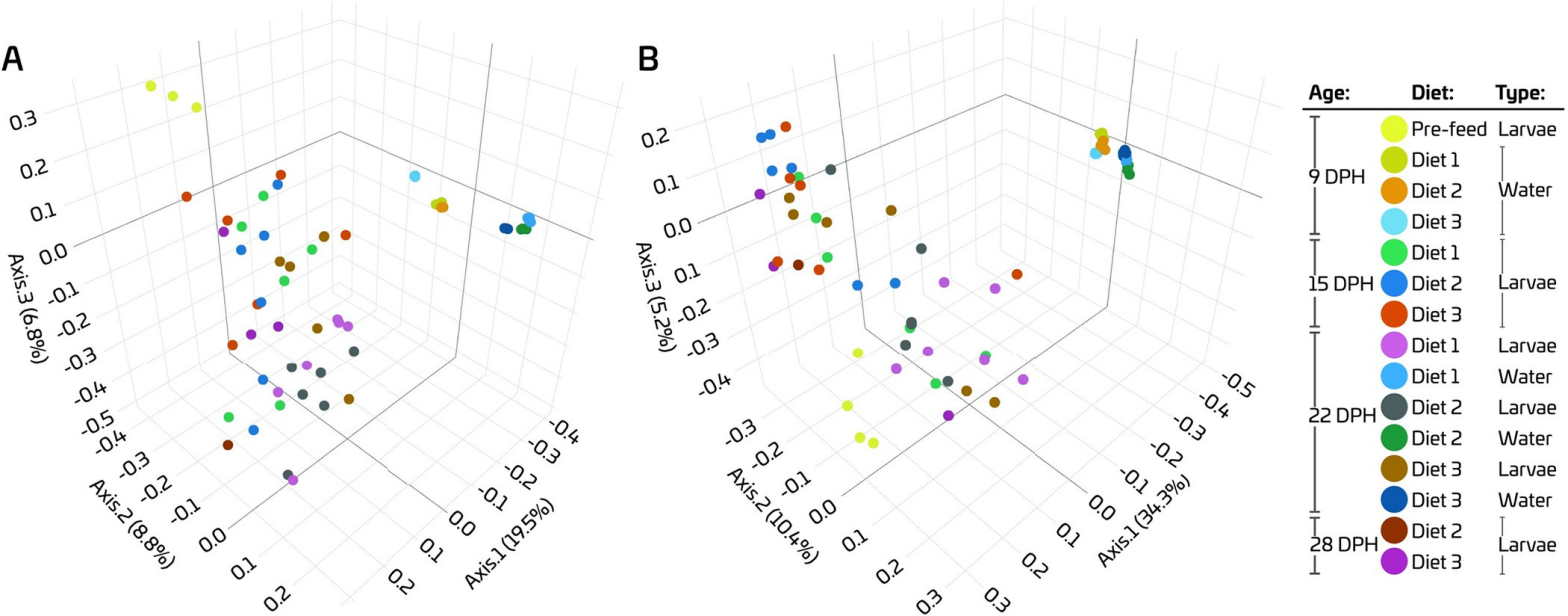
PCoA ordination plots based on Bray–Curtis (A) and Sørensen–Dice (B) dissimilarities for comparison of the bacterial communities of inflowing water and of European eel (*A*. *anguilla*) larvae feeding on different diets. Different colours indicate different samples. Bray-Curtis and Sørensen-Dice are both percent dissimilarities based on relative abundance and presence/absence, respectively.

We assessed the microbial diversity between different diets, as well as over the age by performing PCoA using the Bray-Curtis and Sørensen–Dice distance metrices. The three axes of the PCoA plot based on the Sørensen–Dice distances captured a higher amount of variation (49.9%), compared to the plot based on the Bray Curtis distances (35.1%) (Fig 5). Overall, water samples were clustered tightly, whereas the larval samples showed a wider spread. Discrete grouping of the inflowing water and larval samples can be seenmainly along the axis 1 in both PCoA plots. For the inflowing water, samples were clustered based on the age, mainly along the axis 2 in both PCoA plots. Moreover, on 9 dph, inflowing water samples from the RAS allocated for Diet 3 clustered separately from other two diets in the PCoA plot based on the Bray-Curtis distances, whereas no clear separation was observed in the plot based on Sørensen–Dice distances. However, on 22 dph, no clear separation of the inflowing water samples was observed depending on the diets in PCoA plots based on both indices. Interestingly, samples pre-feeding larvae (at 9 dph) were distinctly clustered from the samples of feeding larvae mainly along the axis 3 especially in both PCoA plots. No clear clustering of larval samples was observed depending on the diets fed both on 15 and 22 dph. On 28 dph, samples were not enough to see the differences in larvae based on the diet they were fed.

PERMANOVA indicated that RASs allocated for three diets yielded different bacterial communities in inflowing water both on 9 and 22 dph (Table 3). Consistently, the larval bacterial community was significantly different from the bacterial community of the inflowing water to the rearing tank in all dietary treatments. Bacterial communities of the larvae were not significantly different between experimental groups, indicating that the diet did not influence the bacterial community composition of the larvae. Regardless of the diet, larval bacterial communities on 9 dph were significantly different from bacterial communities on 15 and 22 dph (Table 4). For the groups fed Diets 1 and 2, bacterial communities on 15 and 22 dph were significantly different based on both dissimilarity measures. However, for the larvae fed Diet 3, bacterial communities on 15 and 22 dph were different based only on Bray-Curtis index. Moreover, for the larvae fed Diet 3, bacterial community on 28 dph was significantly different compared to 9 dph, while being comparable to bacterial communities on 15 and 22 dph. Overall, different bacterial communities in the pre-feeding and feeding larvae were detected indicating that the initiation of feeding influenced the bacterial community composition in the larvae.

**Table 3:** PERMANOVA R^2^ and p-values based on Bray–Curtis and Sørensen–Dice indices for comparisons of bacterial communities between RAS water allocated for different diets, between European eel (*A*. *anguilla*) larvae and RAS water, and between larvae fed different diets at different developmental stages. Significance level was set at <0.05 and significant p values were bolded.

Age	Sample type	Pairwise comparison	Bray-Curtis		Sørensen-Dice	
			R^2^ value	P value	R^2^ value	P value
9 dph	System water	Diet 1 vs Diet 2	0.866	**0.027**	0.537	**0.034**
		Diet 1 vs Diet 3	0.891	**0.025**	0.564	**0.029**
		Diet 2 vs Diet 3	0.889	**0.031**	0.550	**0.019**
	System water vs larvae	Diet 1	0.719	**0.033**	0.735	**0.022**
		Diet 2	0.722	**0.028**	0.721	**0.023**
		Diet 3	0.694	**0.03**	0.718	**0.025**
15 dph	Larvae	Diet 1 vs Diet 2	0.094	0.378	0.129	0.079
		Diet 1 vs Diet 3	0.103	0.289	0.096	0.357
		Diet 2 vs Diet 3	0.096	0.314	0.105	0.186
22 dph	System water	Diet 1 vs Diet 2	0.822	**0.027**	0.552	**0.019**
		Diet 1 vs Diet 3	0.831	**0.027**	0.559	**0.029**
		Diet 2 vs Diet 3	0.856	**0.035**	0.578	**0.022**
	Larvae	Diet 1 vs Diet 2	0.143	0.078	0.137	0.071
		Diet 1 vs Diet 3	0.152	0.074	0.146	0.116
		Diet 2 vs Diet 3	0.178	0.054	0.125	0.153
	System water vs larvae	Diet 1	0.516	**0.003**	0.649	**0.004**
		Diet 2	0.576	**0.006**	0.577	**0.006**
		Diet 3	0.399	**0.007**	0.530	**0.006**
28dph	Larvae	Diet 2 vs Diet 3	0.352	0.500	0.385	0.250

**Table 4:** PERMANOVA R^2^ and p-values based on Bray–Curtis and Sørensen–Dice indices for comparisons of bacterial communities in European eel (*A*. *anguilla*) larvae and RAS water allocated for different diets between different ages. Significance level was set at <0.05 and significant p values were bolded.

Diet	Sample type	Pairwise comparison	Bray-Curtis		Sørensen-Dice		
			R^2^ value	P value	R^2^ value	P value
Diet 1	Larvae	9 DPH vs 15 DPH	0.262	**0.009**	0.242	**0.002**
		9 DPH vs 22 DPH	0.443	**0.009**	0.446	**0.007**
		15 DPH vs 22 DPH	0.165	**0.024**	0.171	**0.014**
Diet 2	Larvae	9 DPH vs 15 DPH	0.277	**0.005**	0.280	**0.005**
		9 DPH vs 22 DPH	0.468	**0.007**	0.362	**0.005**
		9 DPH vs 28 DPH	0.538	0.200	0.505	0.200
		15 DPH vs 22 DPH	0.185	**0.008**	0.169	**0.013**
		15 DPH vs 28 DPH	0.145	0.848	0.181	0.293
		22 DPH vs 28 DPH	0.319	0.156	0.337	0.144
Diet 3	Larvae	9 DPH vs 15 DPH	0.263	**0.008**	0.258	**0.004**
		9 DPH vs 22 DPH	0.321	**0.007**	0.316	**0.006**
		9 DPH vs 28 DPH	0.406	**0.036**	0.353	**0.028**
		15 DPH vs 22 DPH	0.145	**0.008**	0.117	0.14
		15 DPH vs 28 DPH	0.166	0.063	0.149	0.197
		22 DPH vs 28 DPH	0.180	0.151	0.192	0.151
Diet 1	Water	9 DPH vs 22 DPH	0.885	**0.029**	0.498	**0.019**
Diet 2	Water	9 DPH vs 22 DPH	0.894	**0.028**	0.471	**0.027**
Diet 3	Water	9 DPH vs 22 DPH	0.878	**0.029**	0.551	**0.029**

#### 3.2.3 Differential abundance testing

Differential abundance analysis revealed that ASVs of potentially harmful genera were significantly more abundant in the bacterial community of larvae on 22 dph than on 15 dph (Table 5). Moreover, we used the RDP SeqMatch tool for more precise taxonomy based on the DNA sequences of those ASVs. Interestingly, we found that the sequences of ASVs that had significantly higher abundance on 22 dph had high similarity to sequences of potentially harmful bacterial strains (Table 5) regardless of the diet fed. However, in the bacterial communities of the larvae fed Diet 1 and 3, we found significantly higher abundances of ASVs belonging to the potentially beneficial genus *Bacteriovorax* on 15 dph compared to 22 dph. However, AVSs belonging to this genus were not differentially abundant on 15 dph compared to 22 dph, in the group fed Diet 2. Differential abundance analysis revealed that potentially detrimental bacteria were more abundant on 22 dph than on 15 dph, and the abundances of ASVs belonging to the potentially beneficial genus *Bacteriovorax* decreased on 22 dph compared to 15 dph.

**Table 5:** ASVs of potentially harmful genera that were found by DESeq2 analysis to be significantly more abundant (p < 0.05) in larval bacterial communities on 22 dph compared to 15 dph in different experimental groups and nearest matches of the DNA sequences of those ASVs as found by the RDP SeqMatch tool.

ASV ID	Diet fed	Genus	log_2_ increase in abundance	RDP match	S_ab score
ASV 94	Diet 1	*Vibrio*	>21	*Vibrio harveyi*	1.00
ASVs: 133 and 296	Diet 1	*Vibrio*	> 21	*Vibrio alginolyticus*	> 0.92
ASV 154	Diet 1 and 3	*Vibrio*	>21	*Vibrio campbellii*	1.00
ASVs: 15 and 1921	Diet 1 and 3	*Enterococcus*	>7	*Enterococcus faecalis*	> 0.90
ASV 3808	Diet 1	*Enterococcus*	>7	*Enterococcus faecalis*	> 0.90
ASV 96	Diet 1	*Pseudomonas*	>10	*Pseudomonas stutzeri*	1.00
ASV 2691	Diet 1	*Shewanella*	>10	*Shewanella sp*.	1.00
ASV 4	Diet 2	*Lactococcus*	>21	*Lactococcus lactis*	1.00
ASV 127	Diet 2	*Pseudomonas*	>21	*Pseudomonas psychrophile*	1.00
ASV 156	Diet 2 and 3	*Flavobacterium*	>21	*Flavobacterium columnare*	1.00
ASV 1425	Diet 2	*Stenotrophomonas*	>21	*Stenotrophomonas maltophilia*	0.95
ASV 236	Diet 2	*Vibrio*	>7	*Vibrio fluvialis*	0.96
ASV 48	Diet 3	*Micrococcus*	>25	*Micrococcus luteus*	1.00
ASV 55	Diet 3	*Mycobacterium*	>25	*Mycobacterium frederiksbergense*	1.00

### 3.3 Expression of immune and stress-related genes

**Fig 6:**
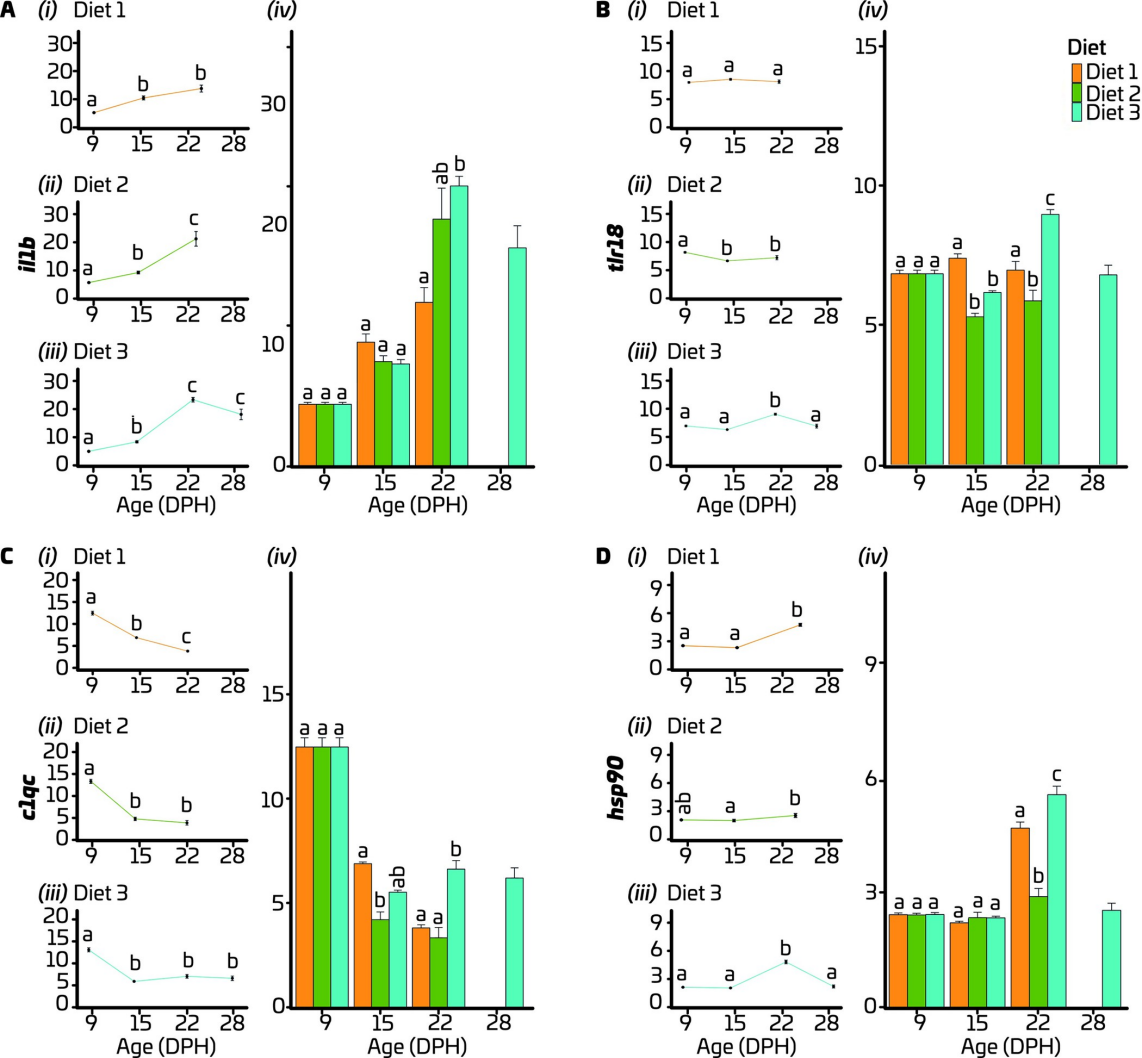
Expression of immune-related genes, *il1b* (A), *tlr18* (B), *c1qc* (C) and stress-related gene, *hsp90* (D) in European eel (*A*. *anguilla*) larvae fed three different diets. All y-axes display gene expression relative to the expression at hatch (0 dph). In each facet, the graphs, i, ii and iii show the effect of age on expression of the relevant gene for each diet, whereas the graph iv shows the effect of diets on gene expression at each age. Values represent means (± SEM), and different lower-case letters represent significant differences (P < 0.05).

Mixed model ANOVAs were used to analyse the relative gene expression data, where the main model variables were age (9, 15, 22 and 28 dph) and treatment (Diet 1, Diet 2, and Diet 3). The initial model tested included an interaction effect between age and treatment. Expression of all selected immune and stress related genes were affected by the age × treatment interaction (p < 0.01). Thus, the model was decomposed into a series of one-way ANOVAs to determine the effect of age for each diet and the effect of diets for each age. In larvae fed Diet 1, relative expression level of pro-inflammatory cytokine, *il1b* was 50% and 62% lower (p < 0.0001) on 9 dph than on 15 and 22 dph, respectively (Fig 6A i). In the group fed Diet 2, *il1b* increased (p < 0.0001) throughout larval development and peaked on 22 dph (Fig 6A ii). In larvae fed Diet 3, this gene was upregulated (p < 0.0001) until 22 dph and then remained relatively stable at high levels (Fig 6A iii). On 15 dph, no effect of diet was found on expression of *il1b*, whereas higher (p < 0.0001) expression of this gene was found in the larvae fed Diet 3 than in the larvae fed Diet 1 on 22 dph (Fig 6A iv).

A pathogen recognition molecule, *tlr18* was expressed at a stable level throughout development in larvae fed Diet 1 (Fig 6B i). In larvae fed Diet 2, expression of *tlr18* was downregulated (p < 0.01) on 15 and 22 dph compared to 9 dph (Fig 6B ii). The expression level of this gene peaked (p < 0.0001) on 22 dph compared to other ages in larvae fed Diet 3 (Fig 6B iii). On 15 dph, a higher (p ≤ 0.0001) expression level of *tlr18* was observed in larvae fed Diet 1 than in larvae fed Diet 2 and 3. On 22 dph, the highest (p < 0.0001) expression level of this gene was detected in larvae fed Diet 3, whereas the lowest (p < 0.001) expression levels were detected in larvae fed Diet 2 (Fig 6B iv).

Expression of the complement component, *c1qc*, was downregulated (p < 0.0001) throughout development in larvae fed Diet 1 (Fig6C i). In larvae fed both Diet 2 and 3, a peak (p < 0.0001) in expression of *c1qc* was observed on 9 dph, after which the expression levels were stable at relatively low levels (Fig 6C ii and iii). On 15 dph, higher (p < 0.0001) expression of this gene was observed in larvae fed Diet 1 compared to larvae fed Diet 2. On 22 dph, *c1qc* was expressed at higher (p < 0.0001) levels in the larvae fed Diet 3 compared to larvae fed Diet 1 and 2 (Fig 6C iv).

The expression of the gene *hsp90*, which is related to cellular stress response and repair mechanism, peaked (p < 0.05) on 22 dph in all larval groups, regardless of the diet fed (Fig 6D i, ii and iii). No differences in expression levels of this gene were detected on 15 dph among the larval groups fed different diets. However, on 22 dph, *hsp90* expression was higher (p < 0.05) in larvae fed Diet 1 compared to larvae fed Diet 2 (Fig 6D iv) and highest (p < 0.0001) in larvae fed Diet 3.

Overall, a gradual increase in the expression levels of *il1b* was detected with the age independent of the diet fed. The highest expression levels of *c1qc* were observed on 9 dph in all experimental groups. Moreover, a significant peak in the expression of *hsp90* was observed on 22 dph independent of the diet fed. The group fed Diet 3 showed significantly higher expression levels of *tlr18* and *c1qc* on 22 dph compared to other two dietary groups. The expression levels of *il1b* in this group was higher than that in the group fed Diet 1, while being comparable to the levels in the group fed Diet 2.

## 4. Discussion

Improvements in assisted reproduction, embryonic incubation and larval rearing have led to a stable production of European eel larvae that are ready to enter the feeding stage. A recent study [18] tested three different experimental slurry-type diets and reported the first successful rearing of European eel beyond the first feeding window. However, two periods of high mortalities were observed, whereas initial mortality occurred shortly after feeding initiation and then again during the period from 20 to 24 dph, indicating the point of no return. In this regard, initiation of feeding, especially with a formulated and slurry-type diet, might lead to deterioration of water quality, due to effusion of nutrients. Consequently, during first-feeding the larvae might be challenged to live in a microbially hostile environment. Here, the innate immune system of larvae might play an important role in protecting the larvae against microbial interference. As such, in the present study, we explored the succession of bacterial communities in larvae and RAS water, but also followed expression patterns of immune and stress-related genes in response to first-feeding diets in European eel. Furthermore, we attempted to disclose the association among bacterial community composition, immune gene expression and larval performance.

Interestingly, several ASVs showing higher similarities to *Bacteriovorax* sp. were found in higher abundances (> log_2_ 22) on 15 compared to 22 dph in the bacterial communities of larvae fed with Diets 1 and 3. *Bacteriovorax* species are well known for their ability to predate on Gram-negative bacteria, by attaching and penetrating through the cell wall to form a bdelloplast and multiply in the periplasmic space of the host bacterium [46, 47]. They play an important role by reducing the bacterial density, while altering microbial community composition through predation and are known to predate on potentially harmful members of Gammaproteobacteria in aquaculture (e.g., bacteria belonging to genera *Vibrio* and *Aeromonas*) [48, 49]. As such, ASVs of the *Bacteriovorax* genus might have played an important role in maintaining a healthy larval bacterial community on 15 compared to 22 dph. We have no data suggesting reasons for the decline in relative abundance of *Bacteriovorax*.

Regarding the characteristic mortality patterns during the first feeding period (as described in [18]), in addition to that the larvae reached the point of no return, the second sharp drop of larval survival could be associated with a shift towards a detrimental larval bacterial community, dominated by potentially harmful bacteria. Interestingly, the analysis of bacterial community composition, conducted in the present study revealed that evenness in the larval bacterial community on 22 dph was lower than on 15 dph, which was noticeable also at the order level of taxonomy. On 22 dph, bacteria of the orders Oceanospirillales, Flavobacteriales, and Alteromonadales have dominated the bacterial community in larvae, regardless of the diet fed. In some cases, these orders have previously been reported to have detrimental effects on marine organisms. For instance, an increase in abundances of bacteria of orders Oceanospirillales and Alteromonadales, which belong to the class of Gammaproteobacteria, have often been associated with stressed and diseased marine invertebrates [50–53]. As such, dominancy of these potentially harmful bacterial orders in the community of 22 dph larvae supports our hypothesis that the second sharp drop in larval survival is linked to a shift towards a deleterious larval bacterial community.

Independent of the diet fed, several ASVs that were more abundant in the larval bacterial communities on 22 dph have been reported as pathogens or opportunistic pathogens. For instance, ASV 94 is similar to *Vibrio harveyi*, which is pathogenic to aquatic organisms [54]. Similarly, DNA sequences of ASVs 133 and 296 matched the sequence of *Vibrio alginolyticus*, frequently reported as the putative agent for outbreaks of vibriosis in cultured fish, such as Gilt-head sea bream (*Sparus aurata*) [55] and grouper (*Epinephelus malabaricus*) [56], and is also associated with abdominal swelling in larvae of several fish species [57, 58]. Moreover, ASV 154 matched *Vibrio campbellii*, which is a pathogen in shrimp hatcheries [59], while ASVs 15, 1921, and 3808 had highest similarity to *Enterococcus faecalis*, an opportunistic fish pathogen, which is one of the causative agents of haemorrhagic septicaemic disease in fish and associated with high mortalities [60–62]. Furthermore, ASV 96 was similar to *Pseudomonas stutzeri*, which is an immerging pathogen in Red Sea seabream (*Diplodus noct*) [63], while ASV 2691 matches *Shewanella* sp., which is also an opportunistic pathogen in fish [64]. Therefore, our hypothesis that the larval bacterial community was dominated by potentially detrimental bacteria on 22 dph and that this could be the reason for parts of the mortality, was supported also at the ASV level.

Considering the inflowing RAS water, a more even bacterial community structure was observed on 22 compared to 9 dph, where the fraction of Vibrionales, which appeared in the RAS allocated to Diet 3, clearly decreased by 22 dph. In aquaculture, several members of the order Vibrionales are known pathogens or opportunistic pathogens for reared finfish, shellfish, and shrimp [65]. In this regard, the ability of RAS to lower the fraction of opportunistic bacteria by developing and maintaining a more even and stable microbial community composition with higher species diversity has previously been described [19]. Moreover, in the present study, we observed that the bacterial communities of the larvae were dissimilar to the bacterial communities of the inflowing water. This might be an indication that even though the inflowing water probably sourced the bacteria for the larval bacterial community, selection inside the rearing tanks played an important role for community assembly [66]. Here, especially due to the atypical feeding behaviour of European eel larvae in captivity, where they dive into a puddle of slurry-type diet, it is important to mention that food-related bacteria might also be substantially driving the larval bacterial community structure. However, further experimentation is required to confirm this.

At the same time, on 22 dph, in all groups of larvae, regardless of the diet, expression of *hsp90* peaked on 22 dph. Expression patterns of heat shock proteins are affected in a wide variety of fish cells and tissues in response to both biotic stressors (such as infectious pathogens) as well as abiotic stressors (such as temperature). Generally, heat shock proteins are constitutively expressed in cells to maintain a number of critical cellular processes relating to protein folding, fidelity and translocation with the task to ensure survival by protecting vital cellular functions [67]. As such, this family of proteins is also commonly referred to as “stress/repair proteins” and an upregulation of *hsp90* has been previously described in several fish species, including the responses of silver sea bream (*Sparus sarba*) to *Vibrio alginolyticus* [68], miiuy croaker (*Miichthys miiuy*) to *Vibrio anguillarum* [69], and marbled eel (*Anguilla marmorata*) to *Aeromonas hydrophila* [70]. Thus, in the present study, the upregulation of *hsp90* observed on 22 dph, might be a response to the presence of potentially harmful bacteria in the larval bacterial community, triggering the activation of the stress/repair mechanism, which might be of key essence, especially considering that the larval immunocompetence is not yet fully developed.

Regarding immunocompetence, generally, the innate immune system recognizes conserved pathogen-associated molecular patterns (PAMPs) through their interaction with specific pattern recognition receptors (PRRs) and can facilitate a direct successful removal of pathogens, e.g. by phagocytosis, or may trigger additional protective responses through induction of adaptive immune responses [71]. In this regard, toll-like receptors (TLRs) are evolutionarily conserved PRRs that play a crucial role in innate immune responses by activation of immune cells that combat invading pathogens [72]. For instance, TRL18 is a member of the fish-specific TLR1 subfamily, reported to directly interact with adaptor proteins and signal downstream to modulate the production of pro-inflammatory cytokines and numerous other immune-related proteins [73]. Cytokines, are small proteins interacting with cells, ligands, and receptors to activate cell-mediated immune responses that aid both, innate and acquired immune system [74]. Here, expression of IL-1β, a pro-inflammatory cytokine, leads to activation of lymphocytes and synthesis of acute phase proteins and thus, activation of the complement system [75]. On the other hand, C1QC is a member of the complement system, aiding several immune functions, including pathogen opsonization, phagocytosis, and inflammatory reactions [76]. In the present study, highest expression levels of the PRR *tlr18* and complement component *c1qc* were detected on 22 dph in the larvae fed with Diet 3. Similarly, expression of the pro-inflammatory cytokine *il-1b* was higher in the larvae fed Diet 3 compared to Diet 1. In other fish species, upregulation of these immune genes has been linked to challenges with pathogenic bacteria or PAMPs [73, 77–81]. As such, the immune components related to pathogen recognition, cellular signalling pathway and complement proteins, important for opsonization, phagocytosis and eventual lysis of harmful microorganisms, which were expressed at higher levels on 22 dph in the larvae fed with Diet 3, potentially indicate a higher immunocompetency, probably assisted in handling the potentially harmful bacteria that dominated the larval bacterial community at this stage, leading to better performance in terms of survival.

Overall, Diet 3 has sustained physiological mechanisms vital for life compared to the other two diets tested, allowing larvae to survive throughout and beyond the first-feeding window [18]. As such, a factor contributing to the higher survival observed in larvae fed Diet 3 might be a higher immunocompetency, which could potentially be supported by the immunomodulatory properties of the included dietary whey proteins, allowing the larvae to better cope with their hostile microbial environment. Whey protein concentrate is considered to be an immune stimulating agent, because it contains bioactive compounds such as α-lactalbumin, β-lactoglobulin, all casein fractions, and lactoferrin (reviewed in [82]). Inclusion of whey protein to replace up to 27.7% of fish meal in Nile tilapia (*Oreochromis niloticus*) fingerling diets has shown to improve performance in terms of growth and survival as well as immune status of fish challenged with *A*. *hydrophila* [83]. Similarly, improved lysosomal and phagocytic activity has been reported in response to inclusion of whey in Barramundi (*Lates calcarifer*) diets [84]. In our study, it is important to mention that an elevated immune response and higher survival were only observed in the larvae fed Diet 3 and not Diet 2, which also included whey proteins, but in much lower levels, indicating that this inclusion level might be insufficient to facilitate the same immunomodulatory effect. Thus, dietary inclusion of whey proteins might be of value, but inclusion levels need to be considered in future.

## 5. Conclusion

To conclude, first-feeding culture of European eel larvae was characterized by a critical period close to the end of the first-feeding window (around 22 dph), which was marked by a sharp drop in survival and an increased activity of the stress/repair mechanism as indicated by elevated expression of *hsp90*. At the same time, the larvae were exposed to a hostile microbial environment, where potentially harmful bacteria dominated the larval bacterial community, indicating that the steep drop in survival observed during the critical period was probably linked partly to detrimental larvae-bacteria interactions. Moreover, the larval group fed Diet 3 that contained whey protein (10%) showed a better immunocompetency on 22 dph, which presumably aided this larval group in handling the potentially detrimental bacteria better than the other two groups, and thus perform (i.e., survival) better after the critical period. The inclusion of whey seems to benefit the European eel larvae; however, inclusion levels need to be optimised. For the first time, the present study reveals the bacterial interference during rearing of the feeding European eel larvae and the significance of larval immunocompetency for sustaining larval survival in a microbially hostile environment.
